# The effect of time–space compression in the Yangtze River Delta region under transportation integration: An accessibility-based analysis

**DOI:** 10.1371/journal.pone.0338912

**Published:** 2026-02-03

**Authors:** Shuo Shang, Haibing Jiang

**Affiliations:** School of Marine and Geographic Sciences, Yancheng Teachers University, Yancheng, Jiangsu, China; Peking University, CHINA

## Abstract

The Yangtze River Delta (YRD) region is one of the areas in China with the highest level of transportation integration, featuring a well-developed high-speed rail network, multimodal transport system, interconnected intercity bus services, and intelligent transportation platforms. These developments have brought about significant effects of time-space compression in the region. Under the effects of time-space compression, the changes in the regional socio-economic spatial structure are worth further exploration. To this end, from the perspective of accessibility, this study explores the time–space compression effect in the YRD region using models such as economic linkage strength (ELS), central city isochronous-ring, daily accessibility (DA), and employment accessibility (EA) indicators. The results show: (1) The time distance between central cities and prefectural-level and lower-tier cities has greatly decreased, resulting in a network-based structure and a trend towards urban system flattening; within 1–2 hours of central cities, the scale of the population, number of cities, and regional coverage area have rapidly expanded, promoting regional integration and urban agglomeration. (2) Intercity time-space compression has significantly increased the total ELS between cities, intensifying the “strong center” economic linkage pattern. The radiation and driving capacity of central cities for the region has notably increased, and the equilibrium of urban economic linkage capabilities has improved. (3) Time-space compression strengthens the “competitive-cooperative effect” of central cities, facilitating the convenient sharing and equalization of high-quality urban public services, but also leading to tensions in the supply and demand of these resources. (4) A high-accessibility region has formed in the “Shanghai-Nanjing-Hangzhou” triangle, where high-accessibility cities possess larger market hinterlands and more employment opportunities, providing significant opportunities for the rise of small and medium-sized cities in peripheral areas. Based on these findings, in the context of time-space compression, these regions should actively build convenient, efficient, and resilient commuting and transfer networks. A networked spatial development strategy should be implemented, establishing efficient regional collaboration mechanisms and public service cooperation systems to promote coordinated regional development.

## 1. Introduction

### 1.1. Background

The *Outline of the Construction of a Strong Transportation Nation* and the *Outline of the National Comprehensive Three-dimensional Transportation Network Planning* point out that by 2035, China will have basically formed a modern integrated transportation system, completed an interconnected transportation network and comprehensive transportation hub system, enabling smooth and convenient intermodal transportation for passengers. The “National 123 Travel Traffic Circle in China” will be essentially established, with metropolitan areas having a 1-hour commute, urban agglomerations accessible within 2 hours, and all major cities in the country covered within 3 hours. The *14th Five-Year Plan for the Development of the Modern Comprehensive Transportation System* in China emphasizes accelerating the integrated development of various modes of transportation.

At the regional level, the *Yangtze River Delta (YRD) Region Transport Higher Quality Integration Development Plan* stresses the need to accelerate the construction of a modern integrated transportation system in the YRD region, aiming to form an integrated transportation infrastructure network by 2025 and essentially create “the YRD on the Rails.” National and local transportation plans guide the integrated construction of high-speed rail, highways, urban rail transit, and urban expressways in the region. At the same time, the *YRD Regional Integration Development Plan* emphasizes deepening cross-regional cooperation, forming an integrated market system and achieving infrastructure interconnection, deep integration of science and technology industries, joint ecological protection, and equitable sharing of public services. Under the guidance of these plans, the impact mechanism of transportation integration on regional integration development has attracted scholars’ attention.

Transportation integration integrates the advantages of various transportation modes as the multimodal transportation systems, which significantly reduces intercity passenger and freight transportation time costs, and achieves the optimal travel time between regions. Driven by transportation integration, the comprehensive transportation network will dramatically bring about regional time-space compression effects. Therefore, the impact of time-space compression under the influence of transportation integration on the development pattern of regional integration and the regional spatial organizational structure in the YRD region deserves extensive and in-depth exploration.

### 1.2. Literature review

The concept of “time-space compression” originated from geographer Janelle’s term “spatial-temporal convergence,” which refers to the phenomenon where transportation technological innovations increase travel speeds and shorten the time distance between cities, making the original spatial distance seem compressed [1]. Based on this, Harvey introduced the concept of time-space compression, which emphasized that the acceleration of the pace of time and space can help to overcome spatial barriers [2]. Time-space compression also was considered as the process in which temporal and spatial experiences change as technologies of transportation and communication advance [3]. Technological innovations in transportation lead to time-space compression, which in turn reduces travel time costs greatly. In regard to commuting trip, there is the notion of constant travel time budget and stable commuting times [4,5], which implies the faster growth in commuting distance with higher travel speed.

Time-space compression due to the high-speed transportation model has a profound impact on business location choices, industrial layouts, tourism spatial structures, and the equalization of public services. Firstly, high-speed transportation infrastructure promotes intercity trade and labor mobility, expanding the market scale of cities along the route and driving the diffusion of employment and industrial activities from central cities to small and medium-sized cities [6–10]. This has a clear knowledge spillover effect, making cities along the route more attractive to venture capital and R&D investments [11], improving R&D output, and transforming these cities into knowledge-intensive industries [12–14]. Secondly, high-speed transportation greatly shortens the time distance between tourist origin and destination, enhancing the attractiveness of scenic spots and increasing competition between similar types of tourist destinations [15]. The high-speed rail effect on regional tourism flows exhibits characteristics such as the Matthew effect, filtering effect, diffusion effect, and overlapping effect [16]. Finally, the opening of high-speed transportation promotes the equalization of public services, with central cities’ public service facilities leading to polarization effects [17].

Accessibility analysis is an important method to explore the time-space compression effect of transportation facilities. For example, isochronous-ring approach from accessibility analysis provides the maps which can describe the changes in the travel time to central cities brought by high-speed transportation routes. Comprehensive transportation accessibility has a significant positive impact on the spatial distribution of manufacturing industries. If there is complementarity between two or more transportation modes, improving transportation accessibility through a comprehensive transportation network can enhance the collaborative effect of transportation and contribute to local economic development [18].

### 1.3. Theoretical foundations

The classical *Central Place Theory* systematically explains the number, size, and distribution pattern of central places from Christaller and Lösch. The central place system is composed of different levels of central places arranged in a hexagonal pattern. Modern *Central Place Theory* posits that the central system is in a dynamic process, and the emergence of modern transportation systems has led to the decline of lower-level central places and a reduction in the number of regional central places [19].

*Regional Spatial Structure Theory* refers to the spatial distribution and combination of various economic activities within a region. During different stages of regional development, the spatial structure of a region presents various patterns, including core-periphery, point-axial, and network-based structures. Under different regional spatial structures, different spatial development strategies are implemented, such as growth pole development strategy, point-axial development strategy and networked development strategy [20].

Lösch’s market location theory suggests that the farther a product is from its source, the higher the transportation costs, which results in higher prices and reduced demand, until at some point the transportation cost becomes so high that the product is no longer saleable. This theory highlights transportation costs as a key determinant in the degree of demand attenuation over distance. When the production price remains constant, a significant reduction in transportation costs can substantially increase market potential.

Palander’s market location theory introduces the factor of spatial competition in business market analysis, stating that market boundaries are constrained by product factory prices and transportation rates. When factory prices are the same, businesses with lower transportation rates can occupy a larger market space [21]. This theory emphasizes the significance of transportation costs in spatial market competition. According to Palander’s market location theory, time-space compression expands the market space of enterprises, increases market proximity levels, and intensifies market competition. In this context, the reduction in transportation costs due to advances in transportation technology enhances product market competitiveness, broadens market service areas, and attracts industries to cluster in locations with superior transportation. This effect is known as the “cost-space convergence” effect [22].

### 1.4. Literature gap

The existing literature provides the scientific basis for our study. But there is still some boundedness. Most recent research has focused on the time-space compression effect of single high-speed transportation modes on urban and regional development. However, research on the time-space compression effect of comprehensive transportation networks on urban and regional economic spatial evolution is relatively underdeveloped. Under the influence of transportation integration, the collaboration between urban rapid transit and regional high-speed transportation will further enhance the regional time-space compression effect. Therefore, there is an urgent need to strengthen research on the “time-space compression” effect of regions under the influence of transportation integration. Based on this, this paper takes the Yangtze River Delta (YRD) region as a case and analyzes the pattern and changes of comprehensive transportation accessibility at the county and district level in order to evaluate the regional time-space compression effect driven by transportation integration construction process. Compared with prior studies, the possible contributions of this paper are mainly analyzing the “time-space compression” effect due to the multimodal transportation system instead of single high-speed transportation model.

The structure of this study is organized as follows. Section 2 details the data sources. Section 3 introduces the measurement methods for time-space compression. Section 4 presents the preliminary empirical results of this study and investigates the effect of time space compression in the context of transportation integration. Finally, Section 5 outlines the key conclusions of this study and offers policy recommendations.

## 2. Data

According to the *Yangtze River Delta Regional Integration Development Plan*, the YRD region includes Shanghai, Jiangsu, Zhejiang, and Anhui, covering an area of approximately 358,000 km². In 2020, the region had a permanent population of 235 million, with 167 million in urban areas. This region boasts a well-developed integrated transportation network, consisting of expressways, general railways (GRs) and high-speed rails (HSRs) as for intercity travel as well as city road, buses and urban rail transit, as for intra-city travel.

Employment and population data, Geographical Information System (GIS) spatial data, and travel impedance by transit are collected to measure the accessibility. Details are discussed as follows.

### 2.1. Employment and population data

Employment and population data is sourced from the *China County Statistical Yearbook* for 2011 and 2021 in S1 File, the statistical yearbooks of each province and city, and data from the Sixth and Seventh National Population Censuses in China. Population data for counties and districts in YRD region focuses on urban population sizes. Employment and population are regarded as the relevant opportunity in the cumulative accessibility measure, which include the employment accessibility (EA) and daily accessibility (DA). Also, population is used to calculate the value of economic linkage strength (ELS) model.

### 2.2. GIS spatial data

GIS spatial data includes geographical boundaries and transportation network data.

This study uses the county-level scale as the primary unit of analysis. The geographical boundaries of each county or district in YRD region are extracted from National Basic Geographic Databases by the National Catalogue Service for Geographic Information (National Geomatics Center of China, 2024). The YRD region encompasses 305 counties and districts in 2022 and their centroids are selected as the origins and destinations for accessibility calculations. Also, the YRD region had 319 county-level administrative units in 2010 and calibrated based on the official administrative division defined by the Ministry of Civil Affairs.

Transportation network data includes the urban roads, expressways, GRs network and stations, HSRs network and stations in the YRD region in 2010 and 2022. On one hand, the transportation network data in 2010 mainly is collected from Institute of Geographic Sciences and Resources, Chinese Academy of Sciences. On the other hand, the road and expressway data mainly come from the OpenStreetMap website in 2022. The HSRs network data in 2022 come from the timetable data of China State Railway Group Co., Ltd. These different transportation networks eventually form the multi-modal travel system.

The base map of YRD region for Figs 3-7 in the article was produced by the research team, which was based on the standard maps with approval numbers GS (2016) 1605 and GS (2020) 3189 downloaded from the Standard Map Service website of the Ministry of Natural Resources of the People’s Republic of China. This base map includes spatial data such as administrative boundaries, city location, the Yangtze River, lakes and railways.

## 3. Methodology

In order to quantitatively investigate the time-space compression effect of transportation integration on regions, we selected indicators and approaches such as intercity temporal distance, central city isochronous-ring, accessibility, and economic linkage strength (ELS) for analysis. Among their indicators, accessibility was initially defined as “the potential of opportunities for interaction [23]. Daily accessibility (DA) and employment accessibility (EA) are chosen as the accessibility indicators in this article, which is measured by counting the number of activities or opportunities available at a given distance from an origin [24]. These accessibility indicators consider two important factors including the travel time between two points and the distribution of the activities. By employing GIS software and natural breaks classification method, accessibility and economic linkage strength (ELS) indicator maps in YRD region for analysis are divided into five levels.

### 3.1. The OD travel impedance matrix

Travel impedance data between origins and destination are the key parameter toward the measure of the follow indicators including the intercity time distance, the central city isochronous-ring and accessibility as well as ELS index. In this article, calculate the shortest travel time between the origin-destination (OD) pairs on the multi-modal travel system by ArcGIS software.

Based on models from relevant studies on the multi-modal transportation accessibility [25], we selected and set transportation network speeds to construct two types of spatial models for shortest travel time between OD pairs in this paper. The 2010 travel impedance model was developed using network analysis in ArcGIS, and the 2022 travel impedance model was developed using a combination of HSRs timetables and road network analysis [25]. Additionally, it is assumed that a 30-minute transfer time in the GRs stations or HSRs stations. The transport transfer mode includes both transfers between trains and roads, as well as transfers between trains.

GIS platforms, network analyst module in ArcGIS and multi-modal transportation network data are used to calculate the shortest time distance between the OD pairs. Additionally, the fundamental Dijkstra’s algorithm is used in the network analyst module. Then the travel impedance between the OD pairs is recorded as corresponding elements in the OD matrix.

The analysis applied accessibility indicators such as isochronous-ring and intercity shortest time distance between the OD pairs. The 2010 model generated the 101,761 OD pairs (a 319 × 319 matrix), while the 2022 model generated 93,025 OD pairs (a 305 × 305 matrix) in S2 File. Then the intercity time distance in the YRD region can be visualized by the OD travel impedance matrix in ArcGIS. Furthermore, we extract shortest time distance value of the central city from OD travel impedance matrix in S3 File and utilize the inverse distance interpolation method to generate the isochronous-ring map of the central city in ArcGIS.

### 3.2. Economic linkage strength model

According to spatial interaction theory and gravity model, the intercity socio-economic linkage strength is proportional to city size and inversely proportional to the distance between cities. Urban spatial interaction reflects the interdependence between urban regions, which is an important indicator of urban spatial development trends, influence range, and functional positioning. A model based on this theory was designed to measure intercity economic linkage strength. ELS model reflects both the diffusion and polarization capabilities of economic centers on surrounding areas and the ability of surrounding areas to accept the economic center’s radiation potential [26]. ELS model is shown in equations (1) and (2). Equation (1) Represents the intercity spatial interaction strength. Equation (2) refers to the Refers to the total ELS within the entire region or the total external ELS of a certain city. The total ELS indicates the external economic diffusion capability and attractiveness of a certain city [27], which evaluate quantitatively the impact of integrated transportation construction on the economic diffusion capability and attractiveness of a certain city in this paper.


$$r_{ij}=\frac{\sqrt{P_{i}}\sqrt{P_{j}}}{D_{ij}^{\alpha }}$$ (1)

$$\begin{array}{c} R=\sum_{i}^{n}R_{i},\;R_{i}=\sum_{j}^{n}r_{ij} \end{array}$$ (2)

Where: *R* is the total ELS for the entire region, *R*_*i*_ is the total ELS of region *i*, *r*_*ij*_ is the ELS between region *i* and region *j*, *P*_*i*_ and *P*_*j*_ are the permanent populations of region *i* and region *j*, *D*_*ij*_ is the time distance between region *i* and *j*, which is used in this study instead of spatial distance. *D*_*ij*_ is the travel time distance between county *i* and *j* in this paper. Because travel time distance can reflect the momentous role of transportation infrastructure, we choose travel time distance rather than spatial distance. According to the related reference [27], set α as 2 in YRD region, α is called the time distance decay coefficient.

### 3.3. Daily accessibility

Daily accessibility (DA) refers to the amount of population or economic activity that can be reached from a certain city within a specific travel time limit, which is useful for calculating the accessibility in business and tourist trips [28]. In the context of the transportation integration, DA represents shown in equation (3) basically the market potential level of a certain city in different period. Therefore, DA is used to evaluates time-space compression impact driven by transportation integration construction on the market size of a certain city’s enterprise and public services in this paper.


$$\begin{array}{c} {DA}_{i}=\sum_{j=\text{1}}^{n}{pop}_{j}\;\text{f}({\text{t}}_{\text{ij}}) \end{array}$$

$$\text{f}(t_{ij})=\left\{ \;\;\;\;\;\begin{array}{c} \;\text{1},\;if\;\;t_{ij}\leq T\;\;\\ \;\;\text{0},\;\;if\;\;t_{ij}\;>T \end{array}\right.\;$$ (3)

Where, *DA*_*i*_ is the daily accessibility index of county *i* in the threshold time, *Pop*_*j*_ is the population size of county *j*, *t*_*ij*_ is travel impedance between the administrative center of county *i* and *j*, where if the time between county *i* and *j* is less than *T* hours, *δ*_*ij *_= 1, otherwise *δ*_*ij*_ = 0. *T* is set to be the travel time threshold. According to the 2-hour accessibility in urban agglomeration of “*National 123 Travel Traffic Circle in China*” target, we compare the variation in 2-hour regional economic cooperation range between 2010 and 2022. So, we set the *T* as 2 hours in YRD region.

### 3.4. Employment accessibility

Employment accessibility (EA) refers to the number of job opportunities available to workers within a given travel time threshold, using a certain transportation mode [24]. EA usually is used to reflect the convenience of residents in obtaining potential employment opportunities in equation (4). This paper applies the EA to investigate the time-space compression impact from transportation integration on employment convenience of urban residents and employment market.


$$A_{i}\;=\;\sum_{j=\text{1}}^{J}P_{j}f\left(C_{ij}\right)$$

$$f\left(C_{ij}\right)=\;\left\{ \;\;\;\begin{array}{c} \text{1},\;\text{if}\;C_{ij}\leq T\\ \text{0},\;\text{if}\;C_{ij}>T \end{array}\right.\;$$ (4)

Where, *A*_*i*_ is the EA for the administrative center of county *i* within the specified time *T*, *P*_*j*_ represents the number of jobs in county *j*, *C*_*ij*_ is the travel impedance between the administrative center of county *i* and *j*, *T* is the travel time threshold, which is set to 60 minutes in this study.

According to the 1-hour commuting duration in metropolitan area of national *“123 Travel Circle” target* in China, we analyzed the changes in the 1-hour metropolitan area commuting duration between different periods. Therefore, the time threshold values for employment accessibility are set at 1-hour.

### 3.5. Sensitivity analysis of model parameters

ELS model is generally derived from the gravity model. To evaluate the impact of the time distance decay coefficient on the values of ELS, this study measures ELS under varying values of the time distance decay coefficient and conducts a sensitivity analysis accordingly. Within the gravity model, the time distance decay coefficient is typically set to 1 or 2 at the regional scale. Referring to the sensitivity analysis approach proposed by the related reference [29], a sensitivity analysis is conducted setting the time distance decay coefficient from 0.6 to 2.4 at an interval of 0.2 in ELS model. To assess the sensitivity of ELS to the time distance decay coefficient, two analyses are conducted: (1) the main statistical indicators including the mean, maximum, minimum, and standard deviation of the ELS in the YRD region under different coefficients, and (2) the correlation analysis to compare spatial disparities in ELS under different coefficients.

**Fig 1:**
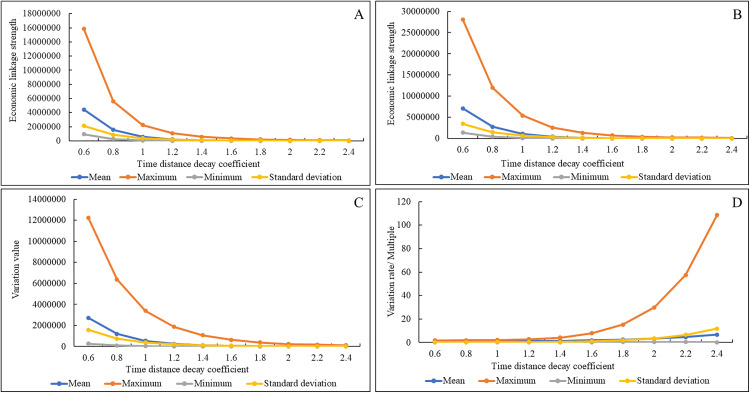
Comparison of the main statistical data of ELS and changes in the YRD region from 2010 to 2022 under different time distance decay coefficients. The main statistical data of ELS is shown in (A) 2010 and (B) 2022. The main statistical data of ELS variation situation from 2010 to 2022 is presented in (C) variation value and (D) variation rate.

Fig 1 presents the main statistics of the ELS and their variation in 2010 and 2022 under different time distance decay coefficients. As the distance decay coefficient increases, both the values of the ELS and their variation exhibit significant declines in key statistical measures, with the gap between maximum and minimum values narrowing sharply. Moreover, the rate of variation in ELS increases progressively with higher coefficient values. These statistical data indicates that the values of ELS and their changes are highly sensitive to the choice of the distance decay coefficient.

**Fig 2:**
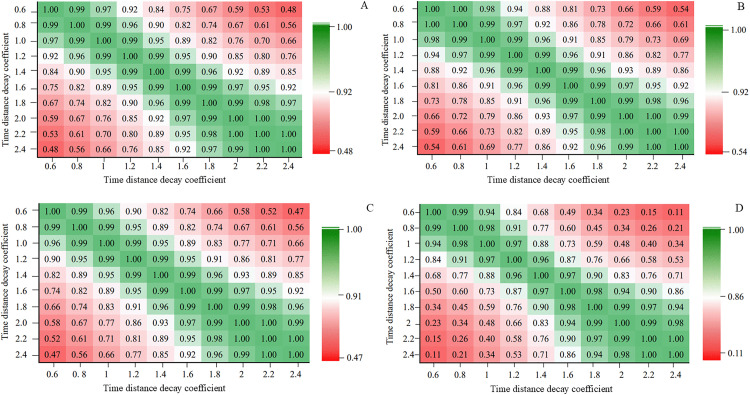
Comparison of the correlation matrices of ELS and changes in the YRD region from 2010 to 2022 under different time distance decay coefficients. **(A)** Correlation matrices of ELS in 2010; **(B)** Correlation matrices of ELS in 2022; **(C)** Correlation matrices of ELS variation value from 2010 to 2022; and **(D)** Correlation matrices of ELS variation rate from 2010 to 2022.

Fig 2 illustrates the correlation coefficients of the ELS and their changes across different distance decay coefficients. Among the 100 correlation coefficients for the values of the ELS and their variation in each group of charts, only 8 fall below 0.6, whereas 30 correlation coefficients fall below 0.6 in the case of ELS’ variation rates. Overall, the correlation analysis suggests that ELS and their changes exhibit high correlations under different distance decay coefficients, implying that despite variations in coefficient values, the spatial patterns of the ELS and their changes under transport integration remain broadly consistent.

In summary, when the time distance decay coefficient is set too low, the estimated values of the ELS and their changes become excessively large, conversely, when it is too high, the values are underestimated. Therefore, setting the time distance decay coefficient at 2 demonstrates relatively strong reliability and rationality in S4 File.

## 4. Results

### 4.1. The shortest time distance comparison of “city Pairs” in the YRD region

Since 2010, the regional transportation system in the YRD region has mainly relied on GRs and highways. The highways and national/provincial roads have expanded coverage, but intercity transportation still faced high time costs. By 2010, the core cities in the region had stronger connectivity, and the connection to smaller cities was relatively weaker. With the introduction of the Shanghai-Nanjing intercity HSRs and Shanghai-Hangzhou HSRs since 2010, and the growing completeness of the transportation network by 2022 (including highways, HSRs, and urban expressways), the intercity connectivity in YRD region has significantly improved.

**(1) The 1-hour “city pair” changes: from core-axial structure to network structure**

**Fig 3:**
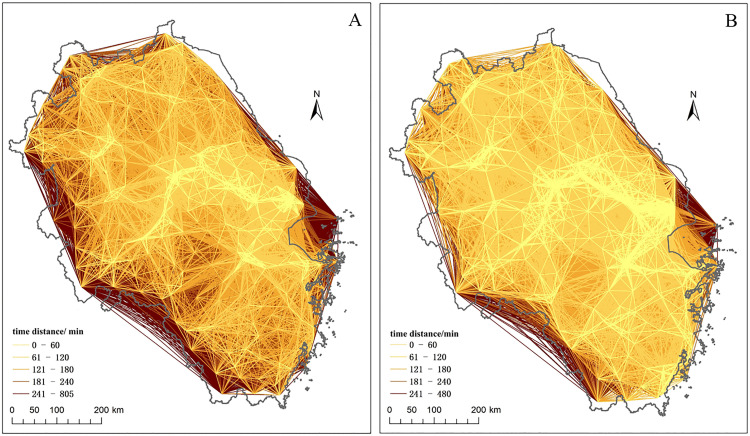
Comparative analysis of the spatial pattern of temporal distance between cities in the YRD region in 2010 and 2022. The spatial patterns of shortest time distance for “city pairs” in the YRD are shown in (A) 2010 and (B) 2022.

Fig 3 and Table 1 show that 1-hour “city pairs” were primarily concentrated around central cities and along the Yangtze River in 2010, forming localized “core” and “axial” patterns. By 2022, the 1-hour city pairs formed a more network-like pattern, covering the entire region. The number of city pairs within 1 hour between prefecture-level cities and surrounding counties greatly increased. The number of 1-hour city pairs doubled and the coverage area of the cities above the prefecture level significantly expanded. In 2010 the average straight-line distance between 1-hour city pairs was 31.9 km, which increased to 46.3 km and increase by 45.1% in 2022.

**Table 1:** Comparative analysis of the shortest time distance between county-level cities in the YRD region in 2010 and 2022.

Time intervals	2010		2022	
	quantity	Proportion	quantity	Proportion
Within 1 hour	2117	2.08%	4303	4.63%
1-2hour	7446	7.32%	14078	15.13%
2-3hour	12932	12.71%	24868	26.73%
3-4hour	17152	16.86%	25358	27.26%
Over 4hours	62114	61.04%	24418	26.25%

In 2010, the accessibility of the Shanghai-Nanjing, Shanghai-Hangzhou and Hangzhou-Ningbo axles and metropolitan areas was relatively superior, with dense 1-hour city pair networks and high efficiency in intercity transport and population movement. According to regional spatial structure theory, the region exhibited a core-periphery and point-axis spatial structure. By 2022, the number of 1-hour city pairs in the five major metropolitan areas of the YRD region increased significantly, covering more widespread and dense areas. The 1-hour commuting zones for metropolitan areas like Shanghai, Hangzhou, Nanjing, Hefei, and Suzhou-Wuxi-Changzhou expanded dramatically. According to the definition of metropolitan scope in relevant national policy documents [30], they are urbanization spaces with a 1-hour commuting circle as their core. The expansion of the five major metropolitan areas is critical for the development of “1-hour commuting circles,” “1-hour economic zones,” and “1-hour living zones” supported by the comprehensive transportation network. It is also common for the 1-hour commuting zones of cities above the prefecture level to extend beyond administrative boundaries, such as in Nanjing, Suzhou, and Wuxi.

Table 1 also shows that the quantity proportion of 2-hour city pairs increased by more than 100% in 2022. In 2010, the amount of the city pairs within 4 hours accounted for about 40%, whereas this share increased to almost 74% in 2022. Conversely, the share of city pairs beyond 4 hours decreased sharply from 61% to 26%, indicating a significant reduction in intercity shortest travel time. In this phase, the region exhibited a network-based spatial structure including core cities, node cities, and high-speed transport networks.

**(2) Significant reduction in shortest time distance between central cities and lower-tier cities leads to flattening of the regional urban system**

Table 2 compares the shortest time distances between medium- and low-level cities to the nearest high-level cities in 2010 and 2022. The results show a substantial reduction in time distance between county-level cities and provincial capitals, with a decrease of around 27% from 2010 to 2022. The time reduction between county-level cities and nearby prefecture-level cities was comparatively lower (around 15.67%).

**Table 2:** Comparative analysis of the shortest time distance from medium- and low-level cities to the nearest high-level city in 2010 and 2022 (min, %).

type	2010年		2022		variation rate	
	average value	Maximum value	average value	Maximum value	average value	Maximum value
Time from prefecture level city to the nearest provincial capital or above city	137.6	254.53	101.45	186.29	26.27	26.81
Time from county-level city to the nearest provincial capital or above city	133.87	394.5	97.09	249.78	27.47	36.68
Time from county-level city to the nearest prefecture level city	38.47	246.93	32.44	205.86	15.67	16.63

Based on modern *Central Place Theory*, the compression of time distance between provincial capitals or central cities and surrounding lower-tier cities significantly alters the time-space relationship, leading to a shift in the regional urban system. Many county-level cities can now directly access provincial capitals or regional central cities, while the influence of prefecture-level cities on surrounding counties weakens. This process flattens the urban system, especially in metropolitan areas where this trend is particularly noticeable. As a result, the service range of central cities in the region expands significantly, and administrative, public service, commercial, and transport hub functions previously held by prefecture-level cities gradually lose their attractiveness. The central cities’ influence increases, leading to a flattening of the urban hierarchy in YRD region.

### 4.2. Spatial characteristics of urban ELS in the YRD region

**(1) Strong increase in internal ELS with enhanced total external linkage capabilities of cities**

The total ELS within the YRD region has significantly increased. In 2010 the total ELS was 2,725,959.9, while in 2022 it reached 8,795,399.7 with a 3.2-fold increase. The average ELS per city rose from 7,506–28,856, nearly quadrupling. The total ELS shows that time-space compression has created more favorable conditions for regional collaborative development.

**(2) Intensification of “strong-center” ELS pattern with central cities’ radiation and drive capacity increasing**

**Fig 4:**
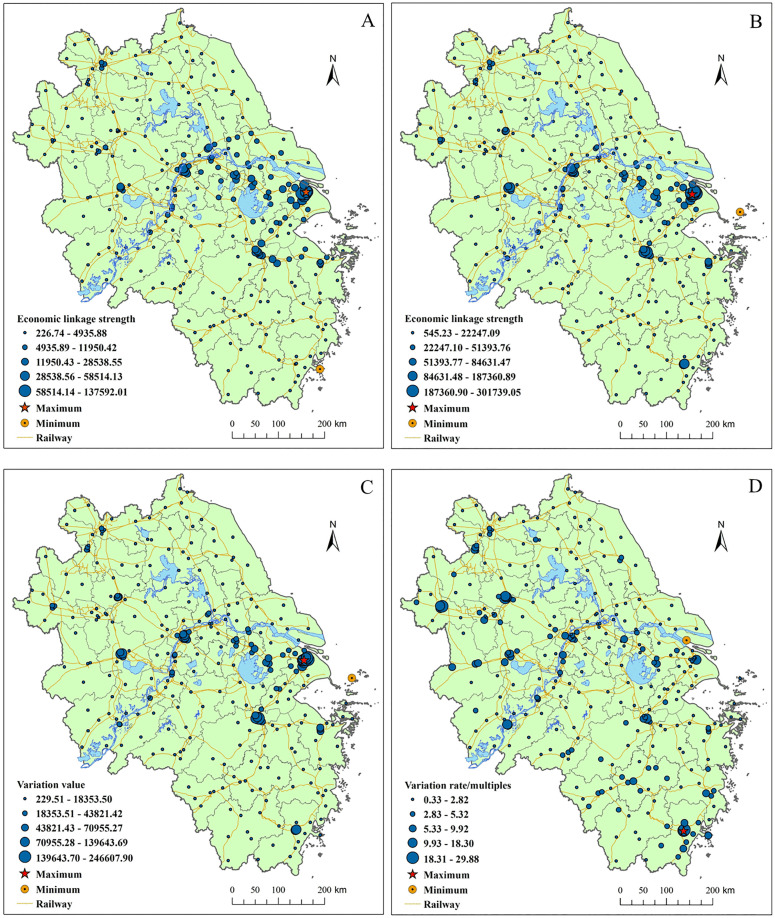
Spatial analysis of ELS and its changes in the YRD region. The spatial patterns of ELS in the YRD are shown in (A) 2010 and (B) 2022. The spatial patterns of ELS variation situation from 2010 to 2022 are shown in (C) variation value and (D) variation rate.

Fig 4 illustrates the prominent increase in the radiation and drive capacity of central cities, improving the overall equilibrium level of the cities ELS in YRD region. In 2010, cities such as Shanghai, Nanjing, and Hangzhou had the highest ELS. By 2022, cities like Shanghai, Nanjing, Hangzhou, and Hefei had the highest ELS. From 2010 to 2022, the ELS in districts like Jing’an, Pudong, Changning, Yangpu, Jianggan, Xuhui, Huangpu, and Yuhuatai increased by over 139,643 units.

The districts with the highest growth rates in ELS include Ouhai, Lucheng, Yingdong, Yuhui, Yingquan, Pudong, Bengshan, Huaishang, Daguan, Yingjiang, Longzihu, Jianggan, and Suixi, with some of these regions seeing increases of over nine times. The coefficient of variation for total ELS across cities dropped from 2.04 in 2010 to 1.68 in 2022, indicating a greater equilibrium in the distribution of ELS across cities. The ELS of peripheral cities grew significantly, with some areas experiencing growth rates exceeding 9 times.

In summary, under the effect of time-space compression, both central and peripheral cities in the YRD region saw substantial improvements in economic and social linkage strength. The overall equilibrium level of ELS between cities has improved, and the leading role of central cities in regional collaborative development has been further consolidated.

### 4.3. Comparative analysis of isochronous-ring of central cities in the YRD region

**(1) The isochronous-rings of central cities extend and diffuse in a “finger-like” and “star-like” pattern along HSRs corridors**

**Fig 5:**
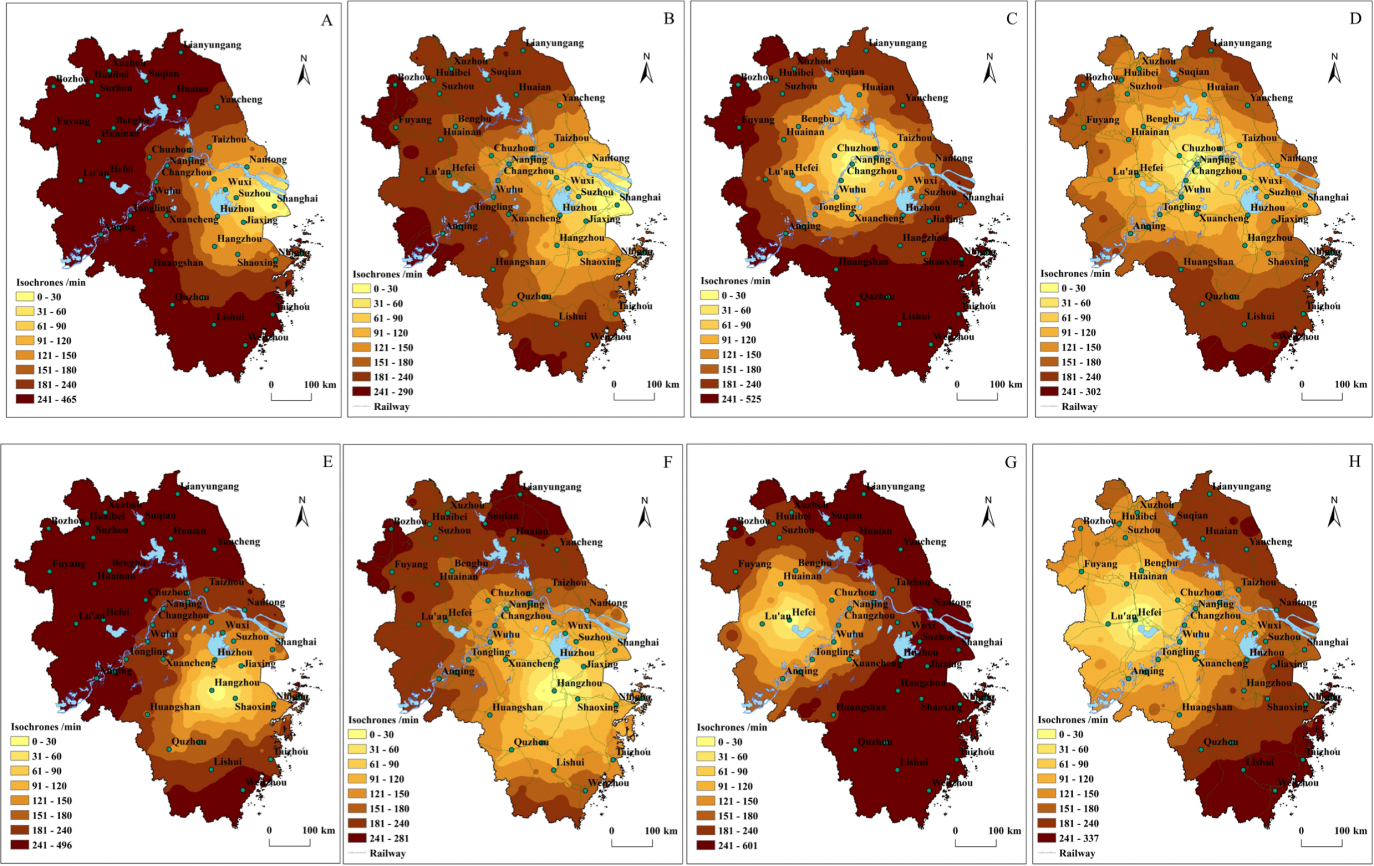
Comparative analysis of isochronous-rings of central cities in the YRD region in 2010 and 2022. The isochronous-rings of central cities in the YRD region in 2010 include **(A)** Shanghai, **(C)** Nanjing, **(E)** Hangzhou and **(G)** Hefei. The isochronous-rings of central cities in 2022 are presented in **(B)** Shanghai, **(D)** Nanjing, **(F)** Hangzhou and **(H)** Hefei.

Fig 5 displays the isochronous-ring of Shanghai, Nanjing, Hangzhou, and Hefei in 2010 and 2022. By comparing the isochronous-rings of regional central cities between the two periods, we find that the isochronous-rings were compact and concentric in 2010, whereas the isochronous-rings were loosely scattered and irregular in 2022, presenting a “star-shaped” extension along the HSRs corridors. HSRs lines such as Beijing-Shanghai, Shanghai-Wuhan-Chengdu, Shanghai-Kunming, and Nanjing-Hangzhou have played a significant role in shaping the spatial form of isochrones. This indicates that HSRs plays a prominent role in time-space compression, which imply that HSRs hubs will increasingly take on a growing function in passenger transport and distribution.

**(2) The 2-hour isochronous-rings of central cities overlap, strengthening the “spatial competition and cooperation effect” between metropolitan areas**

Fig 5, Table 3 and Table 4 show that the 2-hour isochronous-rings of Shanghai, Hangzhou, Nanjing, and Hefei overlap, with Nanjing and Hangzhou covering more than 100 counties and districts, while Shanghai and Hefei cover nearly 90 counties and districts in 2022. The 2-hour radii of Shanghai, Hangzhou, and Nanjing cover around 90 million people. These central cities are located within the 2-hour isochrones of each other. According to Palander’s market location theory, the overlapping of the core hinterland of the central cities significantly enhances the “spatial competition and cooperation effect” between the regional central cities. From the perspective of competition, according to market location theory, spatial compression not only intensifies competition among metropolitan areas in market, talent, capital, and technology but also escalates competition in manufacturing, tourism, and services. From a cooperative perspective, spatial compression provides essential support for political, industrial, academic, and research collaboration between metropolitan areas, allowing the more efficient flow of talent, technology, information, and capital, thus optimizing the allocation of production factors over a broader area. This will lay a crucial foundation for intercity innovation and industrial collaboration in the YRD region, enhancing the region’s international competitiveness in global economic development.

**Table 3:** Comparative analysis of the quantity of accessible cities within 4 hours by central cities in the YRD region from 2010 to 2020.

City	2010				2022			
	Quantity of accessible cities within 1 hour	Quantity of accessible cities within 2 hours	Quantity of accessible cities within 3 hours	Quantity of accessible cities within 4 hours	Quantity of accessible cities within 1 hour	Quantity of accessible cities within 2 hours	Quantity of accessible cities within 3 hours	Quantity of accessible cities within 4 hours
Shanghai	17	56	102	153	22	83	175	282
Nanjing	17	62	134	221	26	116	240	291
Hangzhou	11	35	105	176	22	106	207	280
Hefei	7	29	93	156	11	88	198	267

**Table 4:** Comparative analysis of the population size of accessible cities within 4 hours by central cities in the YRD region in 2010 and 2022 (in 10,000 people).

city	2010				2022			
	accessible population size within 1 hour	accessible population size within 2 hours	accessible population size within 3 hours	accessible population size within 4 hours	accessible population size within 1 hour	accessible population size within 2 hours	accessible population size within 3 hours	accessible population size within 4hours
Shanghai	2322.7896	5038.3473	9515.29	12406.3713	3255.3876	8984.656	14739.7667	21196.0798
Nanjing	913.1495	3437.0866	9330.669	16072.2527	1616.2854	9159.366	18921.0097	22003.9044
Hangzhou	887.4348	2487.7816	8081.925	12635.9017	2098.0248	9887.663	15921.8045	20643.0426
Hefei	570.2466	1682.5768	5093.135	10096.8994	960.2123	4866.355	15043.2041	20617.7314

**(3) The population scale, number of cities and geographic coverage within the 1 and 2hour isochronous-rings of central cities dramatically increase, promoting regional integration and urban integration**

Table 4 shows that in 2010, Shanghai’s population within the 1-hour isochronous-rings was about 23 million, which grew to over 32 million by 2022, with Nanjing, Hefei, and Hangzhou seeing a growth of approximately 100% in their 1-hour accessible population. From 2010 to 2022, the number of counties and districts accessible within 1 hour from Nanjing and Hangzhou increased by 10. In 2010 Shanghai had the highest population within the 2-hour isochrone, with over 50 million people, followed by Nanjing, Hangzhou, and Hefei. In 2022 the 2-hour accessible population of Hangzhou and Nanjing surpassed Shanghai, with the three cities covering between 53.2% and 59.3% of the population in the YRD region. From 2010 to 2022, the number of counties and districts accessible within the 2-hour isochronous-rings of Hangzhou and Hefei increased threefold, while Shanghai and Nanjing’s counties or districts grew by 1.5 times. The dramatic increase in the 1-hour and 2-hour accessible populations of Hangzhou, Nanjing and Hefei reflects the expansion of metropolitan areas and the growing market potential of central cities.

Time-space compression of central and provincial capital cities has led to a significant expansion of their hinterland (Table 5 and Fig 5). From 2010 to 2022, the 1-hour isochronous-rings of Shanghai expanded by 1.57 times, Nanjing by 1.74 times, Hefei by 2.03 times, and Hangzhou by 2.51 times. By 2022, the 2-hour isochrones of Nanjing, Hefei, and Hangzhou covered about 28% of the region, compared to only about 10% in 2010. By 2022, the 3-hour isochronous-rings of Nanjing, Hefei, and Hangzhou covered more than 60% of the region, with Nanjing covering the highest percentage (78.1%), while Shanghai covered 48.5%. Within the 4-hour isochronous-rings, Shanghai, Hangzhou, and Nanjing’s accessible areas covered over 90% of the region, while Hefei reached 87.85%. Time-space compression has expanded the 1-hour living and commuting circles of central cities, accelerating the construction of metropolitan areas and promoting urban integration. This has made cities within metropolitan areas more attractive to external capital, technology and talent, thereby strengthening the central cities’ radiation and service functions for their metropolitan hinterlands.

**Table 5:** Comparative analysis of the accessible area within 4 hours by central cities in the YRD region in 2010 and 2022 (in 10,000 km²).

Time intervals	Nanjing		Hefei		Hangzhou		Shanghai	
	2010	2022	2010	2022	2010	2022	2010	2022
0-30 min	0.31	0.42	0.14	0.51	0.15	0.65	0.36	0.63
30-60 min	0.69	1.32	0.58	0.95	0.59	1.21	0.48	0.69
60-90 min	1.55	2.73	0.98	3.03	0.93	2.36	0.89	1.72
90-120 min	2.22	5.91	1.86	5.59	1.59	5.57	1.55	2.92
120-150 min	3.66	8.35	3.59	6.95	2.71	5.59	2.24	4.41
150-180 min	4.43	9.04	3.31	5.47	3.21	5.87	2.17	6.89
180-240 min	10.06	6.43	6.94	8.74	7.34	11.23	5.71	15.11
Over 240 min	12.64	1.35	18.16	4.32	19.05	3.08	22.16	3.19

**(4) Time-space compression promotes the convenient sharing and equalization of the high-quality public service, but intensifies the “polarization effect” and supply-demand conflicts for such services**

As the hinterland of central cities expands dramatically, the scope of daily activity space for citizens also expands, improving the accessibility of high-quality public services and tourism resources. This increases the coverage of central city public services in education, healthcare, culture, and tourism, facilitating the convenient sharing and equalization of quality public services. It forces metropolitan areas and urban agglomerations to innovate their public service supply modes and improve and implement the integrated policy system for public services, such as promoting policies like cross-regional healthcare services. This will support regional coordinated development.

At the same time, as more citizens share these high-quality public services, the carrying capacity of public services such as healthcare, education, and cultural tourism will face severe challenges, exacerbating supply-demand conflicts. Overcrowding around high-quality public services will become an urban issue that central cities urgently need to address.

The well-developed high-speed transportation network enables residents to access public service facilities and tourist attractions with great efficiency. However, this often results in a rapid concentration of visitors in a short time, which exceeds the maximum carrying capacity of such destinations. For instance, during public holidays, renowned tourist sites such as Shanghai Disneyland, Mount Huangshan, and West Lake in Hangzhou frequently experience visitor flows surpassing their designed reception capacity, which in turn diminishes the overall quality of the tourist experience.

### 4.4. Accessibility analysis of the YRD region

**(1) The market proximity effect increases, and the equilibrium level of city market scale rises**

**Fig 6:**
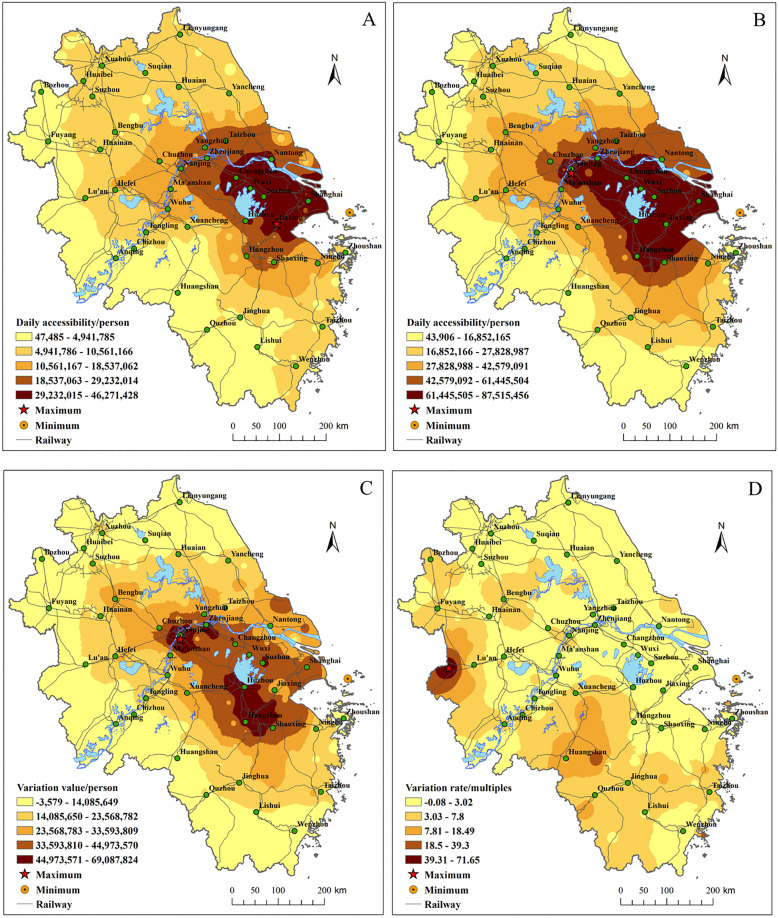
Spatial pattern analysis of DA in the YRD region. The spatial pattern of DA in the YRD is shown in (A) 2010 and (B) 2022. The spatial pattern of DA variation situation from 2010 to 2022 is presented in (C) variation value and (D) variation rate.

The analysis of DA between two periods from Fig 6 and S5 File shows that from 2010 to 2022, DA and its growth areas exhibited a “core-periphery” structure. The high-value region in the “Shanghai-Nanjing-Hangzhou” triangular area expanded, with the DA value in the core area increasing by more than 2 times. The areas with the greatest increase in high-value zones include Nanjing, Hangzhou, Zhenjiang, Jiaxing, and Huzhou. The western and southwestern edge regions experienced a higher growth rate, and the DA values in the peripheral areas showed significant improvement. The equilibrium level of DA increased in YRD region, with the coefficient of variation decreasing by 24.33%. The average DA value increased by 176% in Table 6, which implies that the market size of each city in YRD region has significantly expanded.

**Table 6:** Analysis of the accessibility spatial pattern and its changes in the YRD region.

Indication	2010				2022				Variation rate/ %	
	Minimum/person	Average/person	Maximum/person	coefficient of variation	Minimum/person	Average/person	Maximum/person	coefficient of variation	Average	coefficient of variation
**DA**	47476	13042559	46294704	0.89	43886	36000364	87623213	0.67	176	−24.33
**EA**	4179	241348.2	1399898	1.14	3521	589042.2	2323676	0.84	144	−26.44

The DA analysis indicates that intercity time-space compression has significantly reduced travel costs, effectively expanding the market boundaries for various urban products and services. This has enhanced market potential, generated market proximity effects, and promoted the development of a unified large market. Under the trend of intercity time-space compression, the market scale in the core area of the YRD region continues to grow, forming a massive market that will create the necessary conditions for nurturing strategic emerging industries and future industries in the region. Meanwhile, the consumers in YRD region choose more products and services for a wider range of regions. The following two cases substantiate the above argument.

Case 1. Equalization of healthcare services under the context of transportation integration.

In cities such as Lu’an, Ma’anshan, and Chuzhou in Anhui Province, the HSRs network and urban bus systems have facilitated closer cooperation with major medical institutions in central cities like Nanjing, Shanghai, and Hefei. Leveraging this enhanced connectivity, these localities have established high-level medical collaboration platforms and regularly invited renowned specialists and their teams to provide consultations and perform surgeries at county hospitals. This practice has significantly improved the quality and accessibility of healthcare services in county-level cities.

Case 2. Transportation integration as a driver of high-quality rural tourism development.

The city of Liyang in Changzhou, capitalizing on the Nanjing–Hangzhou HSRs line and intercity bus networks, developed a 365-kilometer scenic route called “No.1 Road”. Since its inauguration in 2018, the number of farm-based restaurants and homestays along the route has tripled within five years. The project has enabled over 100,000 villagers to engage in local entrepreneurship and employment, while between 2018 and 2024, per capita income in the villages along the route increased by more than 10,000 yuan [31], and average collective village revenues rose by 700,000 yuan.

**(2) The spatial spillover effect of employment opportunities has increased, with high-value EA areas distributed along the Shanghai-Nanjing and Shanghai-Hangzhou corridors.**

**Fig 7:**
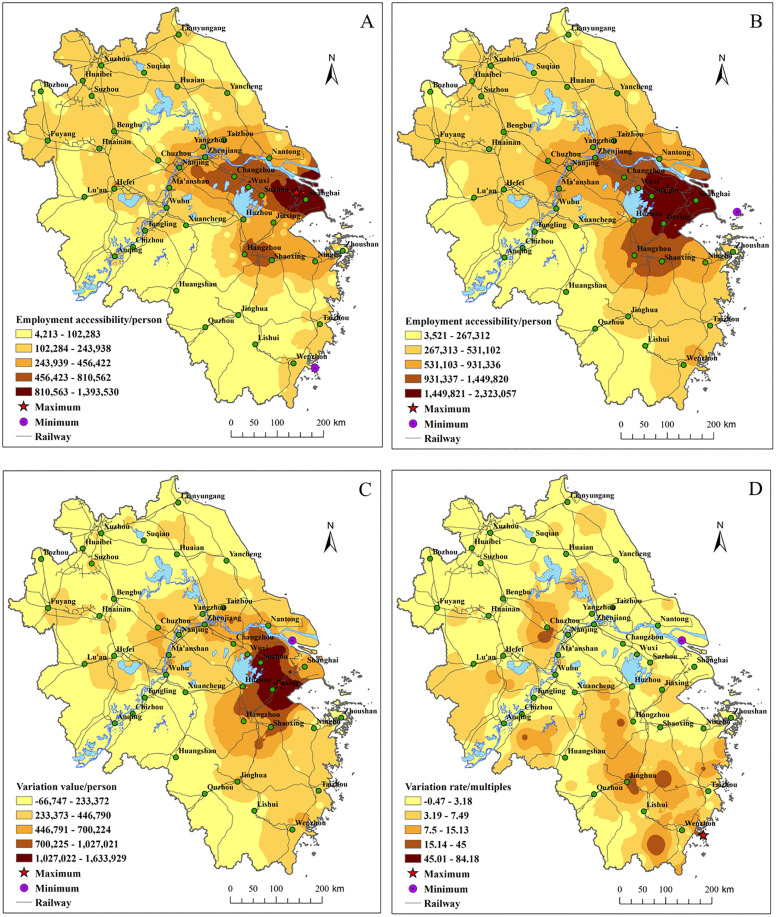
Spatial pattern analysis of EA in the YRD region. The spatial patterns of EA in the YRD are shown in (A) 2010 and (B) 2022. The spatial patterns of EA variation situation from 2010 to 2022 are presented in (C) variation value and (D) variation rate.

From 2010 to 2022, EA in S5 File saw a significant increase with the regional average EA rising by 144% in Fig 7. The equilibrium level of EA improved, with the coefficient of variation decreasing by 26.44%. High-value EA areas expanded from the “Shanghai-Nanjing” axis to both the “Shanghai-Nanjing” and “Shanghai-Hangzhou” axes, forming higher EA corridors along the cities on these axes, attracting a large number of external workers. Suzhou and Jiaxing were high-growth areas for EA, with significant increasement in the counties of Jiangsu and eastern Zhejiang, and the southern Zhejiang region showed notable growth rates of EA. The above values indicate a significant improvement in EA levels in both central and peripheral areas.

The rapid improvement in EA has dramatically expanded the labor market boundaries in the counties along the important traffic axis belt, exacerbating the phenomenon of “job-housing separation” and alleviating the negative impacts of insufficient high quality public services and traffic congestion in suburban areas. It also contributes to reducing the population density in central city areas. Under the effects of transportation integration, intercity time-space compression has contributed to the optimization of regional human resource allocation. This has facilitated the rise of cross-city commuting, expanding the labor supply pool of central cities beyond their local boundaries to encompass the entire metropolitan area. For instance, the Hongqiao Business District in Shanghai demonstrates the broadest coverage of cross-city commuters, with some traveling from as far as Wuxi, Hangzhou, and Nanjing [32]. In summary, time-space compression enhances the spatial spillover effect of “employment opportunities,” promoting labor market integration in YRD region.

## 5. Conclusion and discussion

### 5.1. Conclusion

**Fig 8:**
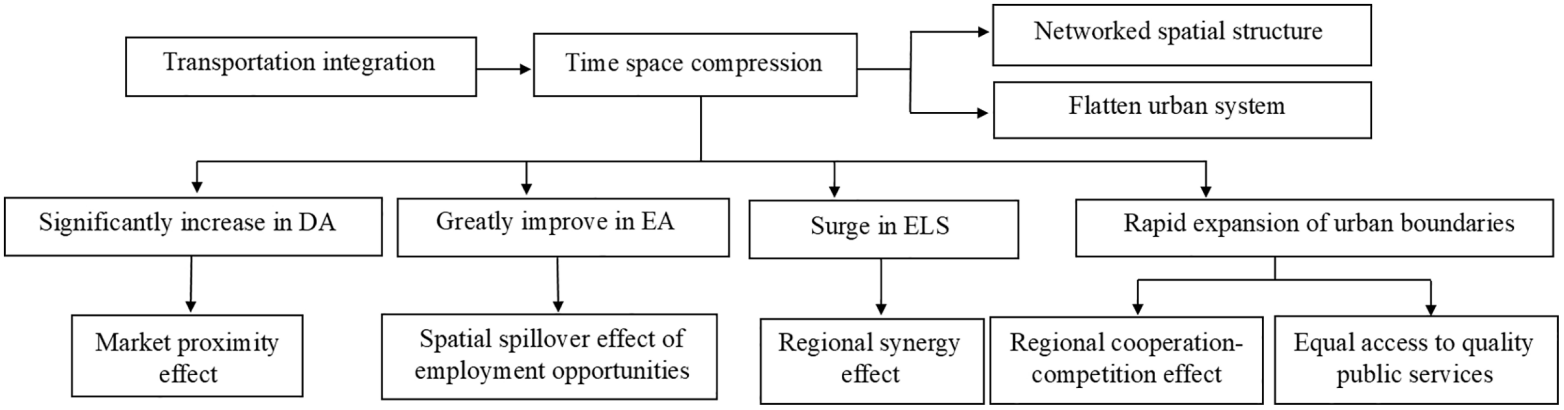
Analytical framework of transport integration and its time-space compression impacts.

In the context of the transportation integration in YRD region, the intercity time-space compression effects in the region include the following in Table 7 and Fig 8.

**Table 7:** Time-Space compression effects and policy recommendations in the YRD region Under Transportation Integration.

Analysis method	Time-space Compression Effect	Policy Recommendations
Analysis on time distance comparison of city pairs	Significant reduction in time distance between central cities and lower-level cities, leading to a flattening of the urban system. The regional spatial structure shifts from a core-periphery and point-axial structure to a network structure.	Utilize the HSRs and expressway networks to implement a network-based spatial development strategy; Build a regional urban system oriented towards transportation integration.
Analysis of urban ELS	Greatly weakens the distance decay effect of intercity interactions, intensifying the strength of intra-regional connections, increasing the dominance of central cities, and improving the support for regional collaborative development.	Establish an efficient regional collaborative development mechanism to form regional coordinated industrial clusters and collaborative innovation communities.
Analysis of center city isochronous-rings	Significantly promotes regional integration and urban integration, expanding metropolitan boundaries and enhancing the “competitive-cooperative effect” among different metropolitans; greatly expands citizens’ daily activity space, promoting the sharing and equalization of quality public services and tourism resources, but also exacerbates the polarization of quality public service resources and supply-demand conflicts; because HSRs plays a crucial role in time-space compression, urban HSRs hubs increasingly gather passengers.	Construct cross-administrative public service cooperation mechanisms, accelerate social security integration and medical insurance coordination, and implement policies for integrated public services, realize the co-construction and sharing of public service resources.
Analysis of DA	Significantly expands the market proximity effect, benefiting the development of a unified large market, and creating preconditions for the cultivation of future industries and strategic emerging industries in the core cities.	The core cities in the YRD region should actively develop future industries and accelerate industrial upgrading.
Analysis of EA	Expands the range of daily commuting across cities, extending the employment space spillover effect, and promoting job-housing separation, which leads to a decrease in population density of the central cities.	Build a convenient and efficient commuting and transfer network, innovate intermodal transportation service models, improve passenger transfer efficiency, and ensure quick cross-city commuting.

Firstly intercity time-space compression doubles the number of “city pairs” within 2 hours, reduces the cost of cross-regional flows of production factors, and shapes a networked spatial structure, causing the urban system to flatten. Secondly it boosts significantly intra-regional ELS level. The average ELS of cities has increased by nearly 4 times, and the total ELS in YRD region has increased by 3.2 times, and the 22 districts of the central cities have the highest ELS, intensifying the strong central economic connection pattern and optimizing conditions for regional collaborative development. This consolidates the central city’s dominant role in collaborative development, providing essential support for regional integration. Thirdly it contributes to regional integration and urban integration in the YRD region, enhancing the “competitive-cooperative effect” between urban agglomerations. The central cities’1-hour isochronous-ring range has expanded by about 2 times, and the their 2-hour isochronous-ring range covers nearly 30% of the YRD region. Fourthly it promotes coordinated regional development and spatial equity in the YRD region. Time-space compression drives the sharing and equalization of public services though it also exacerbates the supply-demand conflict of quality public service resources. Additionally, the equilibrium level of the ELS, DA and EA of the cities in the YRD region has been improved. Fifthly it increased the average DA of cities by 177% and the average EA of cities by 144%, which significantly expands the urban market proximity effect and forms a highest market proximity zone in the “Shanghai-Nanjing-Hangzhou” triangle, and extends the employment opportunity spillover effect and shapes a highest employment accessibility zone along the “Shanghai Nanjing Hangzhou” axis. This promotes the construction of a unified large market and regional market integration in the YRD region. High level accessibility indicates that the region has a large-scale market hinterland and more employment opportunities, which also provides rare development opportunities for emerging peripheral cities.

### 5.2. Discussion

In the context of time-space compression effects from regional transportation integration, this paper proposes the following policy recommendations shown in Table 7 toward the different stakeholders.

Firstly, the rapid expansion of the market radius of high-quality public services and tourism sites challenges the reception and bearing capacity of these services, necessitating the establishment of cross-administrative public service cooperation mechanisms and integrated policies toward the departments of public social services administration, such as inter-regional transportation card usage and medical insurance card settlement.

Secondly the positive effects of time-space compression from regional transportation integration rely on the efficiency of transportation transfers. The construction of comprehensive transportation hubs, integrating HSRs, aviation, and urban public transportation systems for seamless connections, will create extremely high intercity transport efficiency. Additionally, the increased flow through central city transportation hubs and quality public service facilities will face more congestion risks. These tasks and issues need to be addressed by building a convenient, efficient and resilient commuting and transfer network from the urban transportation managers and planners.

Thirdly as for as urban planning managers concern, implementing a network-based spatial development strategy, improving urban network structures, enhancing the capabilities of core network nodes, cultivating new functions for nodes, and improving the transmission efficiency of resources and elements within the network is essential. At the same time, establishing a regionally integrated urban system based on transportation integration and utilizing high-speed transportation networks will enable the consolidation of regional resources, elements, enterprises and economic organizations, which can create the city network system with complementary functions, cooperation and interdependence.

Fourthly intercity time-space compression enables numerous cities to share more markets, labor, talent, and industrial chains, providing the necessary conditions for deepening regional industrial collaboration and innovation. In this context, consumers choose products and services for a wider range of regions, enterprises are also allocating production factors in a larger space to promote the restructuring of supply chain space. Therefore, the municipal economic administration bureaus should follow the development laws of urban agglomerations, make full use of the positive effects of time-space compression, and establish efficient regional collaborative development mechanisms to ensure coordinated regional development. A large market size will provide the necessary conditions for the development of strategic emerging industries and future industries in core areas, promoting industrial upgrading.

Finally, some limitations about transportation integration exist in this study. Due to the lack of actual transfer data among different transportation mode, we adopted a uniform transfer time, which may overestimate the transfer efficiency in some city pairs and consequently thereby affect the calculation results of time-distance for medium- and long-distance city pairs. In future research, a more extensive study focusing on the spatial distribution pattern of actual transfer efficiency and hub efficiency may help to better elucidate the effect of time-space compression in the context of the transportation integration.

## Supporting information

S1 File The dataset of the county and district population and employment in YRD region. (XLSX)

S2 File The dataset of the shortest time distance in YRD region. (XLSX)

S3 File The dataset of the isochronous ring of the central city in YRD region. (XLSX)

S4 File The dataset of the county and district ELS in YRD region. (XLSX)

S5 File The dataset of the county and district DA and EA in YRD region. (XLSX)
